# Male secondary sexual structures and the systematics of the *Thereus oppia* species group (Lepidoptera, Lycaenidae, Eumaeini)

**DOI:** 10.3897/zookeys.520.10134

**Published:** 2015-09-16

**Authors:** Robert K. Robbins, María Dolores Heredia, Robert C. Busby

**Affiliations:** 1 Department of Entomology, PO Box 37012, NHB Stop 105, Smithsonian Institution, Washington, DC 20013-7012 USA; 2 Cra 76A, 9A-34, Apto. 404. Cali, Colombia; 3 7 Countryside Way, Andover, MA 01810-6041 USA

**Keywords:** Dollo’s Law, Loranthaceae, Scent pads, *Thereus brocki*, *Thereus lomalarga*, *Thereus orasus*

## Abstract

The *Thereus oppia* species group includes species with and without a scent pad, which is a histologically and morphologically characterized male secondary sexual structure on the dorsal surface of the forewing. To assess the hypothesis that these structures are lost evolutionarily, but not regained (Dollo’s Law), the taxonomy of this species group is revised. *Thereus lomalarga* **sp. n.**, and *Thereus brocki* **sp. n.**, are described. Diagnostic traits, especially male secondary structures, within the *Thereus oppia* species group are illustrated. Distributional and biological information is summarized for each species. Three species have been reared, and the caterpillars eat Loranthaceae. An inferred phylogeny is consistent with the hypothesis that scent pads in the *Thereus oppia* species group have been lost evolutionarily twice (in allopatry), and not re-gained.

## Introduction

Evolutionary “losses” and “gains” of male secondary sexual structures are being actively documented in the Eumaeini. Evolutionary losses appear to occur when a species is allopatric with its closest relative (Robbins et al. 2012), a result that had been predicted by theory (Phelan and Baker 1987). Evolutionary gains are rare, as appears to be true for most animals (Wiens 2001), especially when the structure was lost previously (Quental 2008). In the three documented lineages in which a new male secondary sexual structure evolved, each clade diversified into more extant species than its sister clade (Robbins and Busby 2015). The Neotropical *Thereus* Hübner (Lycaenidae: Eumaeini) possesses a variety of male secondary sexual structures, for which reason we are beginning to revise the genus systematically. The four primary secondary sexual structures in *Thereus* are forewing scent patches, hindwing scent patches, forewing scent pads, and abdominal brush organs (terminology from Robbins 1991, where these structures are characterized).

*Thereus* was characterized morphologically for 27 species (Robbins 1991). About a third of the species have been reared. With one exception, all were reared from plants in the Santalales, which includes the mistletoes (Robbins 2000, Heredia and Robbins, in prep.). *Rekoa* Kaye and *Arawacus* Kaye were proposed as the closest relatives of *Thereus* based on morphology, and *Rekoa* was later confirmed as its sister genus based on molecular sequences (Quental 2008). *Thereus* contains species with a diverse set of wing patterns and shapes and, as noted, a variety of male secondary sexual traits, which may be the reason that *Thereus* has five junior synonyms (Robbins 2004). Another reason for this lengthy synonymy may be that only one of the three proposed synapomorphies for *Thereus* has been illustrated (Robbins 2000).

The *Thereus oppia* species group, consisting of *Thereus orasus* (Godman & Salvin) and *Thereus oppia* (Godman & Salvin), is distinguished from the remainder of the genus by the presence of scent patches near the costa of the dorsal hindwing and on the inner margin of the ventral forewing (Figs 9–14). *Thereus oppia* also has a “brush” of piliform androconia on the ventral surface of the forewing (Fig. 14), a structure that has not been previously reported in the Eumaeini.

**Figures 1–4.**
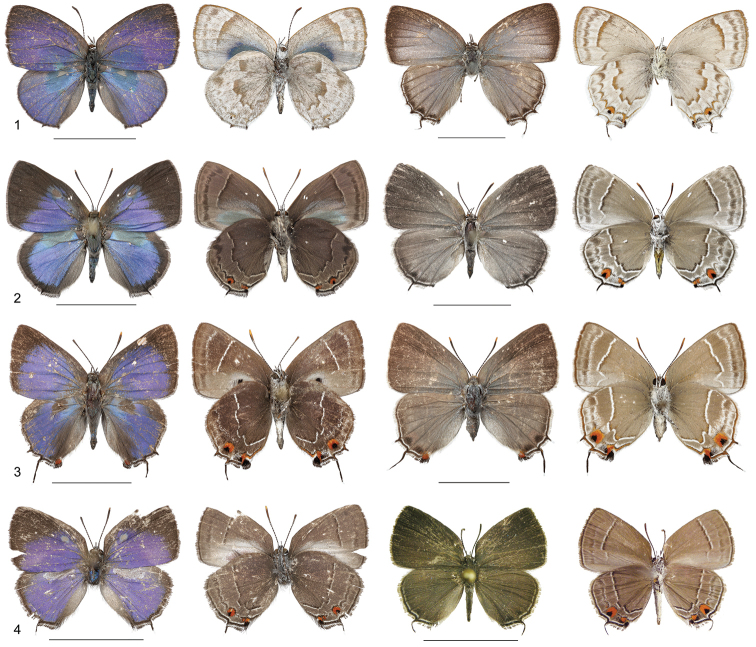
Adults of the *Thereus oppia* species group. Male (left, dorsal wing surface on left) and female (right). **1** *Thereus orasus* ♂ Panama, ♀ Mexico (holotype of *Thecla echinita* Schaus) **2** *Thereus lomalarga* ♂ Colombia (holotype), ♀ Colombia (paratype) **3** *Thereus oppia* ♂ Nicaragua, ♀ Nicaragua **4** *Thereus brocki* ♂ Ecuador (holotype), ♀ Ecuador (paratype). Scale bars: 1.0 cm.

**Figures 5–12.**
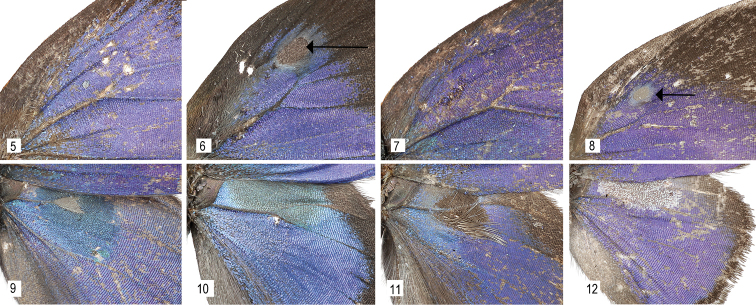
**5–8** Scent pads on the dorsal forewing. **5** *Thereus orasus* (absent) **6** *Thereus lomalarga* (arrow) **7** *Thereus oppia* (absent) **8** *Thereus brocki* (arrow) **9–12** Scent patches on the dorsal hindwing, also showing the convex forewing inner margin. **9** *Thereus orasus* **10** *Thereus lomalarga* **11** *Thereus oppia* **12** *Thereus brocki*.

**Figures 13–14.**
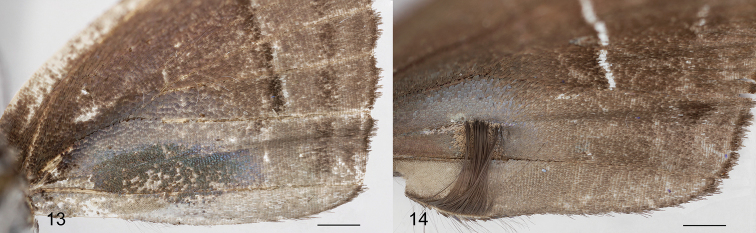
Scent patches on the ventral forewing. **13** *Thereus lomalarga* **14** *Thereus oppia*, showing the erect androconia attached to the inner margin (also in *Thereus brocki*), which occurs in no other Eumaeini. Superficially similar androconia are widespread in tribe Deudorigini. Scale bars: 1.0 mm.

**Figures 15–18.**
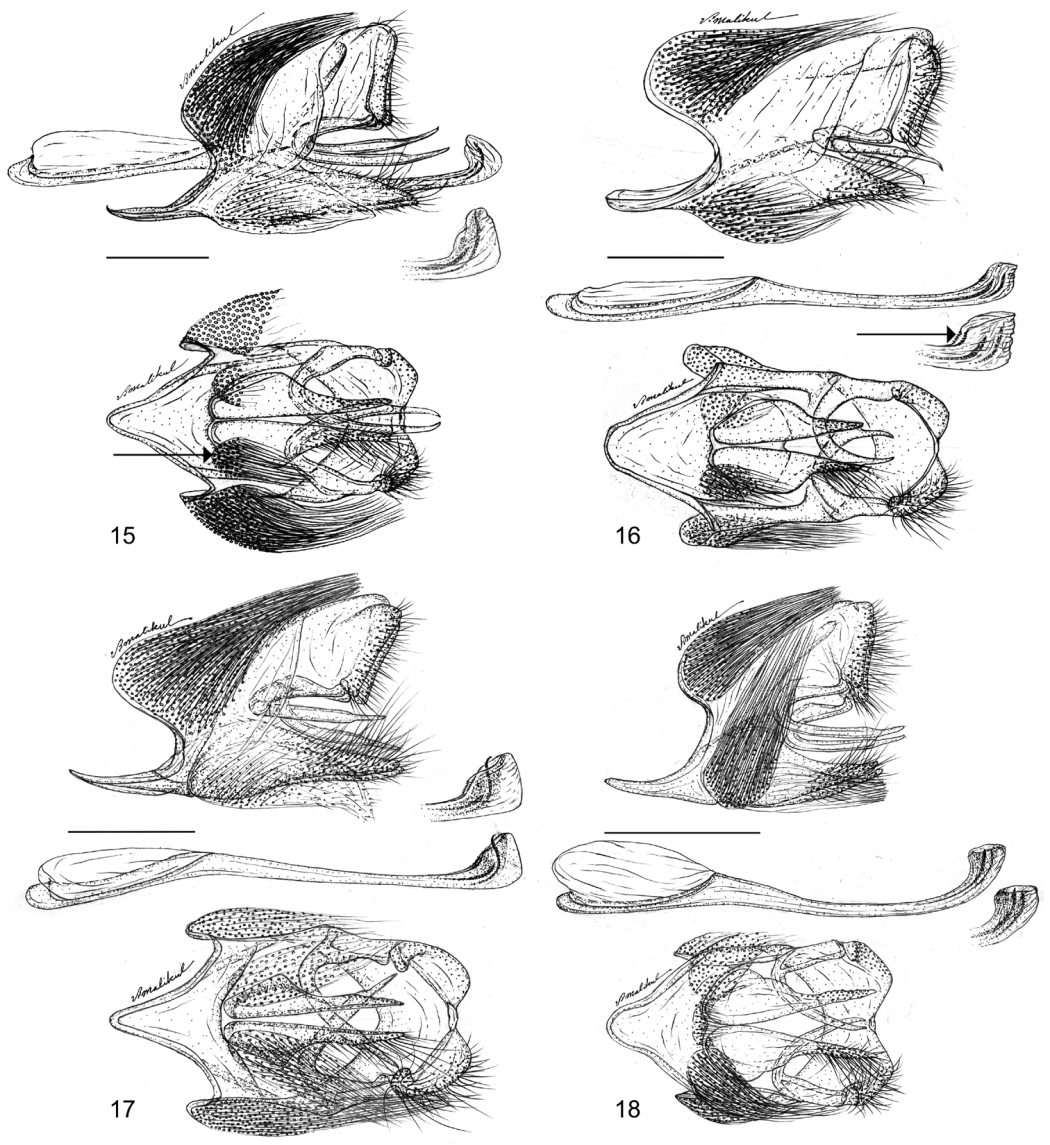
Male genitalia of the *Thereus oppia* species group. Lateral view of capsule and penis (top) with penis tip enlarged and ventral view (bottom). Posterior of insect to the right **15** *Thereus orasus* (arrow points to ventral brush organ) **16** *Thereus lomalarga* (arrow points to position of small teeth) **17** *Thereus oppia* **18** *Thereus brocki*. Scale bars: 0.5 mm.

**Figures 19–22.**
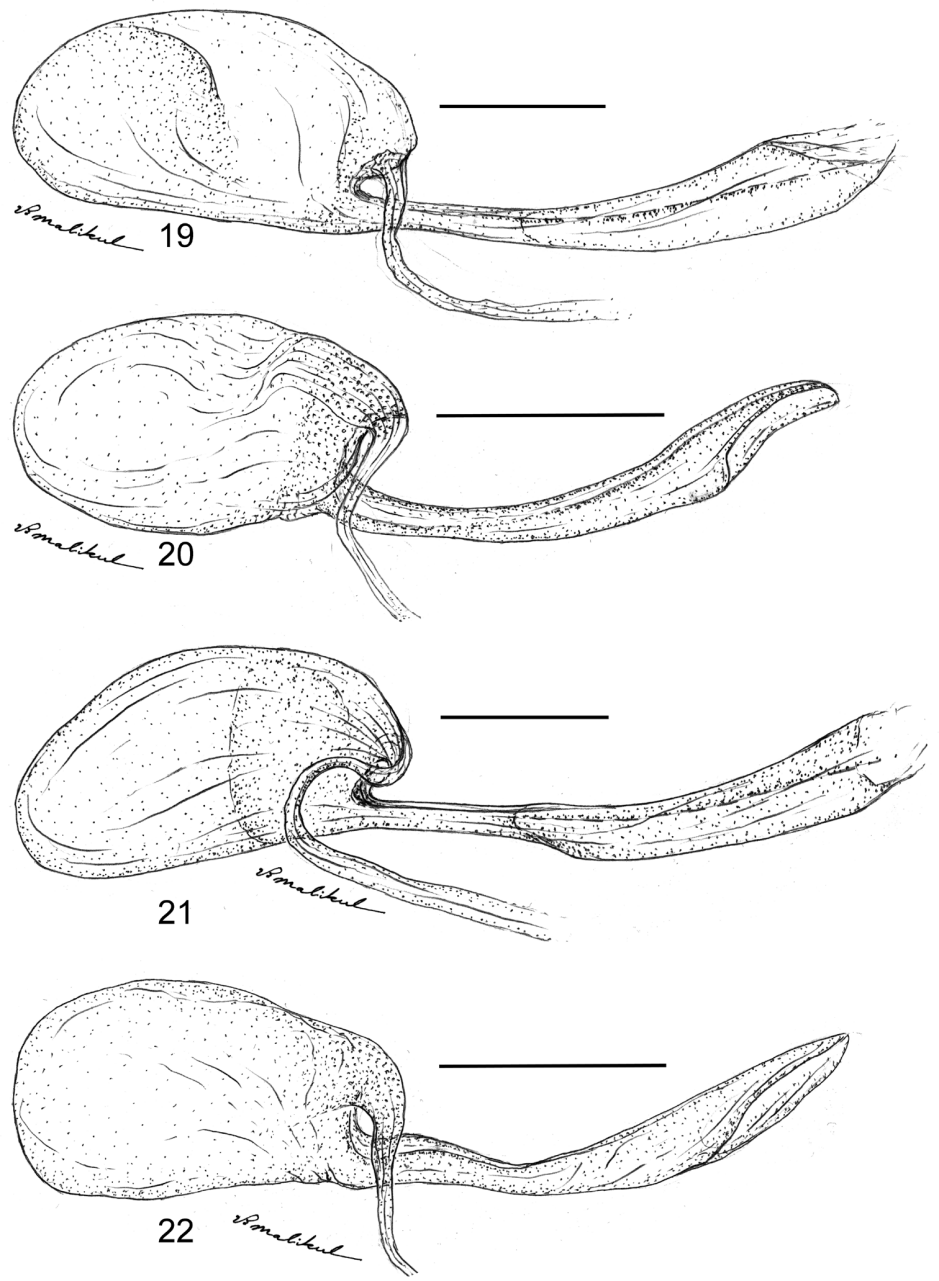
Female bursa copulatrix of the *Thereus oppia* species group. Dorsal (top) and lateral view of the ductus copulatrix. Posterior of insect to the right. **19** *Thereus orasus* **20** *Thereus lomalarga* **21** *Thereus oppia* **22** *Thereus brocki*. Scale bars: 0.5 mm.

**Figures 23–24.**
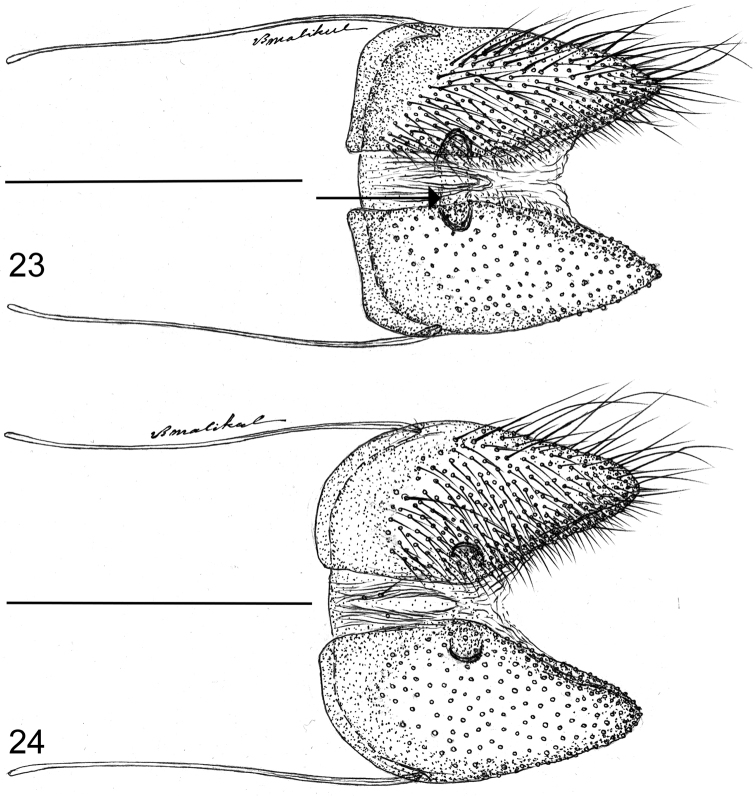
Female papillae anales in ventral aspect showing sclerites that characterize *Thereus* (arrow). Posterior of insect to the right. **23** *Thereus lomalarga* **24** *Thereus brocki*. Scale bars: 0.5 mm.

Another two *Thereus* species have been discovered with scent patches similar to those of the *Thereus oppia* species group (Figs 10, 12, 13). One of these species has a ventral forewing androconial “brush”, and both share virtually indistinguishable male and female genitalic structures with *Thereus orasus* and *Thereus oppia* (Figs 15–24). For these reasons, we add them to the *Thereus oppia* species group. We have reared one of the newly discovered species in Colombia and are documenting its life history (Heredia and Robbins, in prep.).

Two species of the *Thereus oppia* species group lack scent pads on the dorsal surface of the forewing and two possess scent pads (Figs 5–8). Given previous results (Wiens 2001, Quental 2008), we predict that the scent pad was lost evolutionarily once or twice in the *Thereus oppia* species group. However, if the two newly discovered species that possess scent pads were phylogenetic sisters (cf. Discussion), this result would be consistent with the re-evolution of a scent pad, which would represent the first such documented case in the Eumaeini.

One purpose of this paper is to illustrate the proposed traits that characterize *Thereus* and to provide a brief overview of the biology of the genus. Another is to delimit the *Thereus oppia* species group and to provide names for the two unnamed species in this group. These names are needed for the phylogenetic analysis and for the publications of the life history of one of them. The third purpose is to propose a preliminary phylogenetic hypothesis for the *Thereus oppia* species group to assess whether male scent pad re-evolution is likely to have occurred in this species group.

## Materials and methods

The species level taxonomy of the *Thereus oppia* species group is based on an analysis of variation among 121 pinned specimens from various museum and private collections, as noted below. Species accounts include notes, when relevant, on nomenclature, history, morphological variation, elevation, seasonality, and behavior. Males and females were associated by similarity of ventral wing pattern and distribution. In one species, rearing confirmed the association in a species with sexually dimorphic wing patterns. Geographic distributions of the new species are mapped. Labels on holotypes are recorded verbatim with brackets used for information not explicitly noted on the labels and for descriptions of the labels. Otherwise, months are abbreviated by their first three letters in English. Citations for original descriptions can be found in Lamas et al. (1995). Biogeographic zones follow Brown (1982), who partitioned the forested lowland continental Neotropics into the Transandean Region, Amazonian Region, and Atlantic Region. Many male eumaeines perch in “territories” at certain times of day to wait for receptive females to fly through and “defend” these areas by flying at other males that enter the territory (e.g., Powell 1968; Alcock and O’Neill 1987; Cordero et al. 2000). Recorded times from our fieldwork for “territorial” behavior are standard time at that locality.

Morphological characters for the phylogenetic analyses are utilized because DNA sequences are currently available only for *Thereus oppia*. Standard references for morphological terminology are Comstock (1918) for insect wing veins; Klots (1970) and Robbins (1991) for lepidopteran genitalia as modified for the Eumaeini; Robbins (1991) and Robbins et al. (2012) for male secondary sexual structures; and Snodgrass (1935) for all other morphological structures. To quantify nudum extent, the number of segments from the antennal tip to the first segment with a complete ventral row of scales was counted (Robbins 1991).

Four species are recognized in the *Thereus oppia* species group (Table 1), two being newly described. The first outgroup for the phylogenetic analysis is *Thereus cithonius* (Godart), which shares small teeth on the dorsal tip of the penis with two species in the *Thereus oppia* species group (Figs 16, 17). The second outgroup is *Thereus ortalus* (Godman & Salvin), which shares a relatively straight vinculum strut in lateral aspect and a slender “crescent” shaped dorsal cornutus with the *Thereus oppia* species group (Figs 15–18).

**Table 1.** Morphological characters and their states in the *Thereus oppia* species group.

1. Male shape of forewing inner margin (0) straight, (1) convex.
2. Male ventral forewing iridescent blue (0) absent, (1) present.
3. Male dorsal forewing dark border (0) a marginal line, (1) present.
4. Ventral forewing postmedian line shape (0) relatively straight, (1) gently curved.
5. Orange spot on dorsal hindwing anal lobe (0) present, (1) absent.
6. Male dorsal hindwing costa with an iridescent sheen (0) absent, (1) present.
7. Female hindwing tail length (0) same length as male, (1) longer than male.
8. Basal edge of ventral hindwing postmedian line (0) a dark line, (1) a broad orange-brown band.
9. Male ventral forewing scent patch (0) absent, (1) present.
10. Male ventral forewing brush of erect scales (0) absent, (1) present.
11. Male dorsal hindwing with gray-charcoal androconia (0) absent, (1) present and iridescent, (2) present, but not iridescent.
12. Male dorsal forewing scent pad at vein udc (0) present, (1) absent.
13. Male dorsal hindwing scent patch (0) without piliform setae, (1) with piliform setae.
14. Shape of ventral cornutus in male genitalia penis (0) shaped like a crescent moon, (1) posteriorly thickened.
15. Length of male 8^th^ abdominal tergum (0) about 1.1 mm, (1) about 1.7 mm or longer.
16. Ventro-lateral processes of male genitalia vinculum (0) present, (1) absent.
17. Teeth on subterminal dorsal penis of male genitalia (0) absent, (1) present.

Seventeen characters were coded (Table 1), and their states for each ingroup and outgroup species were recorded (Table 2). We searched exhaustively for shortest trees using the implicit enumeration option of TNT software (Goloboff et al. 2008) to derive a most parsimonious cladogram. To test the assumption of equally weighted characters, implied weighting was performed over a range of values for the parameter K (1, 10, 50, 250, and 1000).

**Table 2.** Character matrix for the *Thereus oppia* species group.

Taxa	1	2	3	4	5	6	7	8	9	10	11	12	13	14	15	16	17
*Thereus cithonius* outgroup	0	0	1	0	0	0	0	0	0	0	0	0	0	1	1	0	1
*Thereus ortalus* outgroup	0	0	1	0	0	0	0	0	0	0	0	0	0	0	0	1	0
*Thereus oppia*	1	0	1	0	0	0	0	0	1	1	2	1	1	0	0	1	1
*Thereus brocki*	1	0	1	0	1	0	0	0	1	1	2	0	0	0	0	1	0
*Thereus lomalarga*	1	1	1	0	1	1	1	0	1	0	0	0	0	0	0	1	1
*Thereus orasus*	1	1	0	1	1	1	1	1	1	0	1	1	0	0	0	1	0

To assess scent pad evolution in the *Thereus oppia* species group and to avoid potential circular reasoning, we repeated the analyses with Character 12 (presence or absence of a dorsal forewing scent pad) omitted. Characters were then mapped on trees with WinClada software (Nixon 2002) with the unambiguous changes option.

Specimens cited in this study are deposited in the following collections (abbreviations where available from Evenhuis (2013)).

BMNH The Natural History Museum [formerly British Museum (Natural History)], London, United Kingdom.

IAVH Instituto Alexander von Humboldt, Villa de Leyva, Boyacá, Colombia.

MUSENUV Museo de Entomología de la Universidad del Valle, Cali, Colombia.

MUSM Museo de Historia Natural, Universidad Nacional Mayor de San Marcos, Lima, Peru.

RCB Private Collection of Robert C. Busby, Andover, MA, USA.

UCRC Entomology Research Museum, Department of Entomology, University of California, Riverside, California, USA.

USNM National Museum of Natural History, Smithsonian Institution, Washington, DC, USA.

## Systematics

### Thereus

Taxon classificationAnimaliaLepidopteraLycaenidae

Hübner, [1819]

#### Type species.

***Papilio lausus* Cramer**

#### Diagnosis.

**Figure 25.**
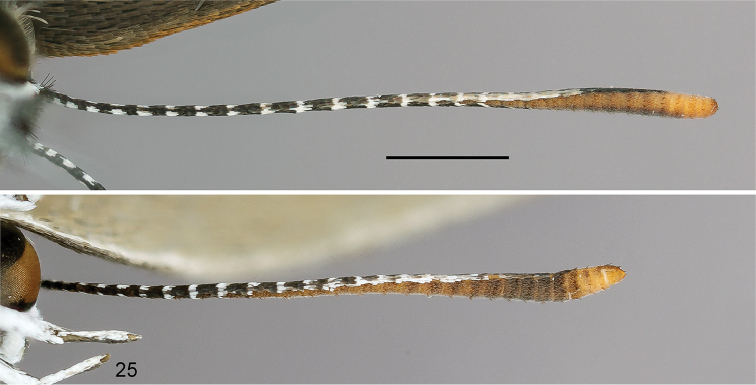
Male (top) and female antennae of *Thereus lomalarga* in ventral aspect showing nudum extent. The male has 14 nudum segments in contrast to 21 nudum segments in the female. Scale bar: 2 mm.

Robbins (1991) characterized *Thereus* by (1) a pair of sclerotized invaginations on the membrane attached to the ventro-lateral sides of the papillae anales (Figs 23–24, figured in Robbins 2000), (2) a pair of ventro-lateral brush organs (often inconspicuous) in addition to the pair of dorsal ones (Figs 15–18), and (3) the number of antennal nudum segments (as defined in the methods section) is sexually dimorphic, being greater in females than that in males by five or more segments (Fig. 25). The second trait is lacking in *Thereus pseudarcula* (Giacomelli), suggesting that this species is the phylogenetic sister to the remainder of the genus. Interestingly, it is the only *Thereus* species that appears to be a subtropical endemic. During this study, we found an instance in which sexual dimorphism of antennal nudum length was four segments—not five or greater—so the generic diagnosis is modified accordingly.

Robbins (1991) noted that *Thereus* contained 27 species, but more than a decade later, 31 were listed, of which nine were undescribed and two had questionable generic placement (Robbins 2004). Bálint (2005) proposed a new specific name, and we now recognize 35 species, of which eleven are undescribed and two have questionable generic placement. This paper begins the task of recognizing species groups, which facilitates the description of new species, and of determining the generic placement of the species with questionable generic placement.

#### Nomenclature.

Robbins (1991) accorded *Thereus* Hübner priority over *Molus* Hübner and synonymized *Noreena* K. Johnson, MacPherson & Ingraham. Subsequently proposed names *Solanorum* Johnson, *Timokla* Johnson, Kruse & Kroenlein, and *Pedusa* d’Abrera were synonymized later (Robbins 2004).

#### Distribution.

*Thereus* occurs throughout the Neotropics from northern Mexico to Uruguay and Argentina. *Thereus lausus*, *Thereus cithonius*, and *Thereus ortalus* range widely from Mexico to southern Brazil, but distributions of species are otherwise more restricted. Approximately 2/3 of the species in the genus occur in the Amazon Region, as demarcated by Brown (1982).

#### Habitat.

Most species inhabit wet lowland forest, with only a few exceptions. *Thereus gabathana* (Strand), *Thereus wojtusiaki* Bálint, *Thereus orasus* (Godman & Salvin), and an undescribed species are montane endemics, and *Thereus pseudarcula* is subtropical. The widespread *Thereus cithonius* occurs in a great variety of habitats, from wet forest to very dry deciduous forest and from sea level to 2,000 m elevation.

#### Biology.

Heredia and Robbins (in prep.) summarize the food plant records for the genus, almost all of which belong to the plant family Loranthaceae (mistletoe). As noted in this paper, males of some species set up mating territories in the morning before 09:30 hours while others set up territories in the early afternoon.

### *Thereus oppia* species group

**Diagnosis.** The four members of the *Thereus oppia* species group possess the proposed synapomorphies of *Thereus* and are distinguished by a convex inner margin of the forewing (Figs 9–12), a scent patch on the dorsal forewing covering the basal part of cell r_s_-M_1 _(Figs 9–12), and a scent patch on the ventral surface of the forewing (Figs 13–14). No other *Thereus* species possesses any of these traits. They also have genitalic structures that are indistinguishable, or nearly so (Figs 15–24).

**Male secondary sexual organs** (Figs 5–18). There are four distinct kinds of male secondary sexual organs in the *Thereus oppia* species group.

(1) Two of the four species have a scent pad on the dorsal surface of the forewing located at the basal origin of veins r_3_ and M_1 _(Figs 5–8). Scent pad histology and morphology have been detailed (Thomas 1893, Robbins 1991, Robbins et al. 2012). Scent pads occur only in the Eumaeini and Tomarini, but no case is known in which a scent pad was lost evolutionarily and then regained.

(2) A scent patch on the dorsal surface of the hindwing centered at the base of cell r_s_-M_1_ occurs in all four species of the *Thereus oppia* group (Figs 9–12), but in no other *Thereus* species. The blue and gray (in *Thereus orasus*) androconia are iridescent in *Thereus orasus* and *Thereus lomalarga* (Figs 9–10). The androconia are gray to black in *Thereus oppia* and *Thereus brocki* (Figs 11–12). There are also piliform shaped androconia in *Thereus oppia* (Fig. 11, noted in Godman and Salvin 1887–1901). Superficially similar kinds of scent patches occur widely in the Eumaeini, such as *Allosmaitia* (Clench 1964) and *Lathecla* (Robbins and Busby 2015), but not in other *Thereus* or *Rekoa*.

(3) A scent patch on the ventral surface of the forewing located between the inner margin and the cubital vein (Figs 13–14). Again, superficially similar scent patches occur widely in the Eumaeini, but not in other *Thereus* or *Rekoa*. In *Thereus oppia* and *Thereus brocki*, there are also erect piliform setae that attach to the inner margin (Fig. 14). The tips of these setae are evident in *Thereus oppia* (Fig. 3, underside of male) and can be seen on the left underside of the male in *Thereus brocki* (Fig. 4). Superficially similar setae occur in the tribe Deudorigini (Eliot 1973: 403), where they are almost universal, but are unreported in other Eumaeini or in any other Theclinae, so far as we are aware. Further, the erect setae in Deudorigini are not associated with a scent patch on the ventral forewing, as in *Thereus oppia* (the light tan scales under the tips of the setae – Fig. 14).

(4) All *Thereus* species have a pair of dorsal and a pair of ventral brush organs (Figs 15–18). Brush organs are bundles of hollow setae attached to the membrane connecting the male genitalia vinculum to the posterior 8^th^ abdominal segment (Eliot 1973). They have a chamber at the anterior end, presumably containing a secretory cell (Robbins 1991). The only other Eumaeini with four brush organs are two species of *Chalybs* Hübner, a genus that is unrelated to *Thereus* (Quental 2008).

**Male genitalia** (Figs 15–18). There is little interspecific variation in the male genitalic structures in the *Thereus oppia* species group. All males of the *Thereus oppia* group possess minute teeth on the vesica inside the penis, but there are also minute teeth on the external ventral tip of the penis of *Thereus lomalarga* and *Thereus oppia* (Figs 16–17). Otherwise, we cannot distinguish the species based on genitalia.

**Female genitalia** (Figs 19–22). The ductus seminalis arises dorsally from the posterior end of the ductus bursae. Signa are absent, but occasionally vestigial remnants can be observed. Although shape and size of the ductus bursae are variable, as illustrated, this variation does not distinguish species.

**Figure 26.**
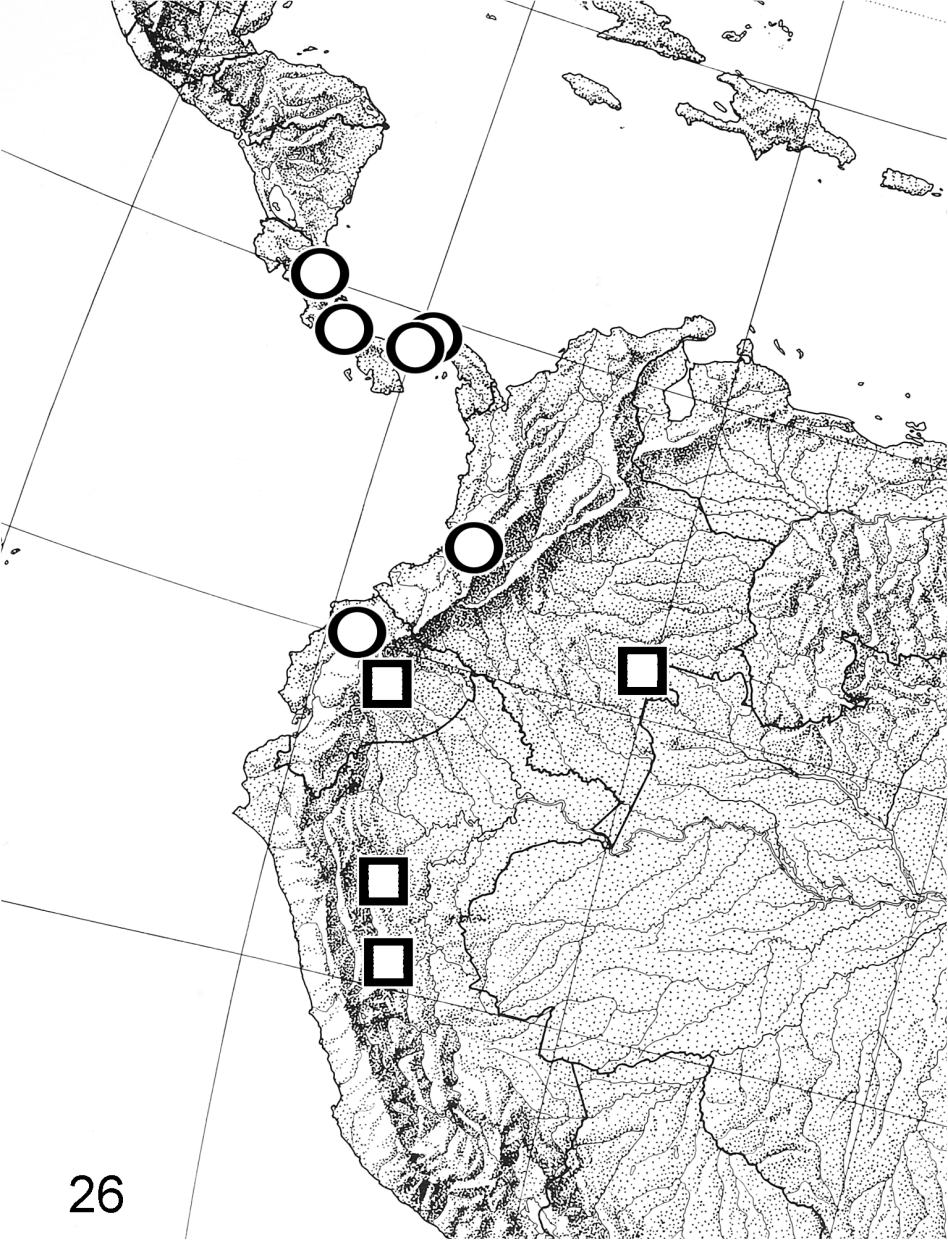
Geographic distribution of *Thereus lomalarga* (circles) and *Thereus brocki* (squares).

**Distribution and habitat** (Fig. 26). Members of the species group occupy the Transandean and Amazon Regions, as outlined in Brown (1982). *Thereus orasus* is a montane endemic, but the others occur in lowland and lower montane forest.

**Biology.** Three of the four species have been reared from Loranthaceae (see below). Male behavior is recorded for *Thereus lomalarga* and *Thereus oppia*.

### Thereus orasus

Taxon classificationAnimaliaLepidopteraLycaenidae

(Godman & Salvin, 1887)

Figs 1 , 5 , 9 , 15 , 19

#### Diagnosis.

*Thereus orasus* differs from other members of the *Thereus oppia* group by having a gray ventral ground color, not brown (Figs 1–4). The male is also unique in having no dorsal forewing brown border (except for some marginal black scales) and the gray part of the dorsal hindwing scent patch is restricted to the basal part of cell r_s_-M_1 _(Fig. 9).

#### Nomenclature.

Robbins (2004) synonymized *Thecla echinita* Schaus (Fig. 1, female type in USNM) with *Thecla orasus* (male holotype in BMNH) because they share a similar ventral wing pattern and occur in the same habitats and have the same distribution. We have examined both types.

#### Distribution and habitat.

*Thereus orasus* is an uncommon species that is recorded from montane habitats from central Mexico (Colima and Veracruz) to those of western Panama (Chiriquí) at elevations from 1100 to 1800 m.

#### Caterpillar food plant.

Greg Ballmer collected a larva on 30 Aug 1988 at El Jabalí, 13 mi NE Comala, Colima, Mexico, at 1100–1200 m. The caterpillar was eating *Struthanthus condensatus* Kuijt (Loranthaceae). An eclosed adult female and its pupal case are deposited in UCRC. The mistletoe plant was growing on coffee and was identified by Kuijt.

### Thereus lomalarga

Taxon classificationAnimaliaLepidopteraLycaenidae

Robbins, Heredia & Busby sp. n.

http://zoobank.org/39501F5C-16C9-437D-874F-74722CEF1AB6

Figs 2 , 6 , 10 , 13 , 16 , 20 , 23 , 25

#### Type material.

**Holotype**: ♂ (Fig. 2). [printed on white paper] COLOMBIA: Valle del Cauca/Cali, Pance, Loma Larga/1200m, 3°19'N/76°34'W/1 April 2011/Leg. M.D. Heredia. [printed label on red paper] Holotype/*Thereus lomalarga*/Robbins, Busby, & Heredia. [printed white barcode label] Instituto Humboldt/Colombia/IAvH-E-146988. Deposited IAVH.

**Paratypes** (32♂, 41♀). **Costa Rica**. 1♀ Turrialba, 2,000 ft, 13 Jul 1965 (USNM). **Panama**. Canal Area. Paraíso, Cerro Luisa, 4 Feb 1979 (2♂ USNM), 16 Feb 1979 (1♂ USNM), 1 Mar 1979 (1♂ USNM), 4 Mar 1979 (1♂ USNM), 10 Mar 1979 (1♂ USNM). Pedro Miguel, Chiva Chiva Road, 14 Jan 1979 (1♀ USNM). Panama Province. Cerro Campana. 1500 ft/500m. 3 Jan 1965 (1♀), 26 Jan 1966 (1♀ USNM), 28 Jan 1980 (1♀ USNM). 850 m. 23 Feb 1979 (1♀ USNM). Chiriquí Province. Potrerillos. 3600 ft. 27 Dec 1965 (1♀USNM), 28 Dec 1965 (2♀ USNM), 27 Dec 1965 (1♀USNM), 1 Jan 1966 (2♀ USNM), 28 Jan 1966 (2♀ USNM), 29 Jan 1966 (1♀ USNM), 2 Feb 1966 (1♀ USNM), 19 Feb 1966 (1♀ USNM), 5 Mar 1966 (1♀ USNM). **Colombia**. Valle del Cauca, Cali. Pance, 3000 ft, 14 Jan 1985 (1♀ USNM)). Loma Larga, 1200m. 3°19'N/76°34'W. 15 May 2009 (1♂ MUSENUV).18 Nov 2010 (1♂ USNM, IAvH-E-112219). 5 Dec 2010 (1♂ MUSENUV). 10 Dec 2010 (1♀ MUSENUV). 13 Jan 2011 (1♀ USNM, IAvH-E-112207). 15 Mar 2011 (1♀ MUSENUV). 17 Mar 2011 (1♀ MUSENUV). 19 Mar 2011 (1♀ MUSENUV). 31 Mar 2011 (1♀ MUSENUV). 1 Apr 2011 (1♂&1♀ MUSENUV). 2 Apr 2011 (1♂ MUSENUV). 5 Apr 2011 (1♀ MUSENUV). 6 Apr 2011 (1♀ IAVH, IAvH-E-146990). 24 Apr 2011 (1♂ IAVH, IAvH-E-146982). 21 May 2011 (1♂ USNM, IAvH-E-146983). 29 May 2011 (1♂ MUSENUV). 30 May 2011 (1♂ IAVH, IAvH-E-146987). 1 Jun 2011 (1♂ USNM, IAvH-E-146985). 15 Jun 2011 (1♂ MUSENUV). 17 Jun 2011 (1♂ MUSENUV). 21 Jun 2011 (1♀ IAVH, IAvH-E-146986). 23 Jun 2011 (1♂ MUSENUV). 27 Jun 2011 (1♀ MUSENUV). 28 Jun 2011 (1♀ IAVH, IAvH-E-146984). 14 Jul 11 (1♂ MUSENUV). 23 Jul 2011 (1♂ MUSENUV). 17 Dec 2011 (1♀ MUSENUV). 31 Dec 2011 (1♀ USNM, IAvH-E-146989). 20 Feb 2012 (1♂ MUSENUV). 23 Feb 2012 (1♀ MUSENUV). 3 Mar 2012 (1♂ MUSENUV). 4 Mar 2012 (1♂ MUSENUV). 6 Mar 2012 (1♂ MUSENUV). 12 Mar 2012 (1♂ MUSENUV). 3 Apr 2012 (1♂ MUSENUV). 15 Apr 2012 (1♂ MUSENUV). 19 Apr 2012 (1♂ MUSENUV). 22 Apr 2012 (1♀ MUSENUV). 29 Aug 2012 (1♂ MUSENUV). 25 Jun 2014 (1♀ MUSENUV). 4 Jul 2014 (1♂ MUSENUV). 3 Jul 2014 (1♂ MUSENUV). **Ecuador**. Pichincha, 10 km Celica-Sardinas Road, 0°11.6'N, 79°00.8'W, 550-775 m, 27 May 2008, (1♀ RCB); 7 km Pacto-Guayabillas Road, 0°09.0'N, 78°48.9'W, 1600m, 18 Jun 2014, (2♀ RCB); 5 km Nanegal- García Moreno Road, 0°09.2'N, 78°39.4'W, 1375–1700m 21 Jan 2015, (1♀ RCB); 24 May 2008, (1♀ RCB).

#### Etymology.

**Figure 27.**
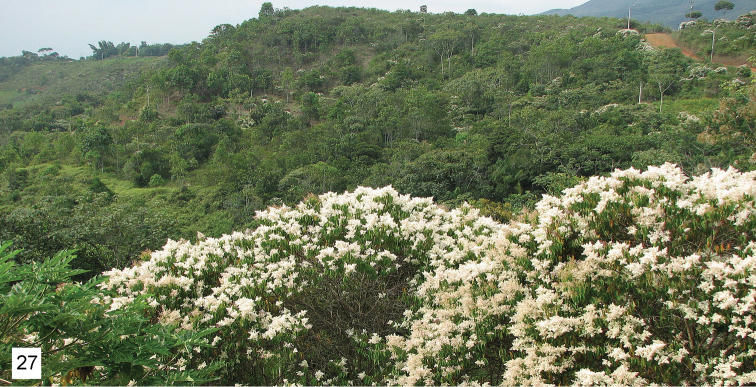
Natural succession forest in Loma Larga, the type locality of *Thereus lomalarga*, with flowering *Miconia minutiflora* (Bonpl.) DC.

This species is named for Loma Larga, a housing development on the outskirts of Parque Nacional Natural Farallones de Cali. Loma Larga has had an ecological and conservation policy for about 15 years that has designated a substantial plot of land for natural forest regeneration (Fig. 27) in contrast to cow pasture. The name is a noun in apposition.

#### Type locality

(Fig. 27). The type locality is naturally regenerated forest in Loma Larga.

#### Diagnosis and description.

*Thereus lomalarga* belongs to *Thereus* because it possesses the synapomorphies of the genus (Figs 16, 23, 25). It belongs to the *Thereus oppia* species group (Figs 2, 10, 13). It shares a dorsal forewing scent pad with *Thereus brocki* (Figs 6, 8), but differs in having an iridescent scent patch on the dorsal hindwing and in lacking erect piliform setae on the inner margin of the ventral forewing (Figs 2, 10, 13). The male has evident teeth on the dorsal tip of the penis (Fig. 16), which distinguishes it from *Thereus orasus* and *Thereus brocki*. The wing pattern, androconia, genitalia, and antennae are illustrated (Figs 2, 6, 10, 13, 16, 20, 23, 25). Mean forewing size of males is 11.67 mm (sd = 0.52, N = 23) and of females is 11.34 mm (sd = 0.65, N = 16).

#### Variation.

Expression of the female orange-red spot at the anal lobe of the ventral hindwing between vein Cu_2_ and the inner margin varies from a fused double spot, as in Fig. 2, to completely absent. The ventral ground color varies from gray to brown. The postmedian line on the ventral hindwing varies slightly in shape from that in Fig. 2 to that of *Thereus brocki* in Fig. 4.

#### Distribution

**Figure 28.**
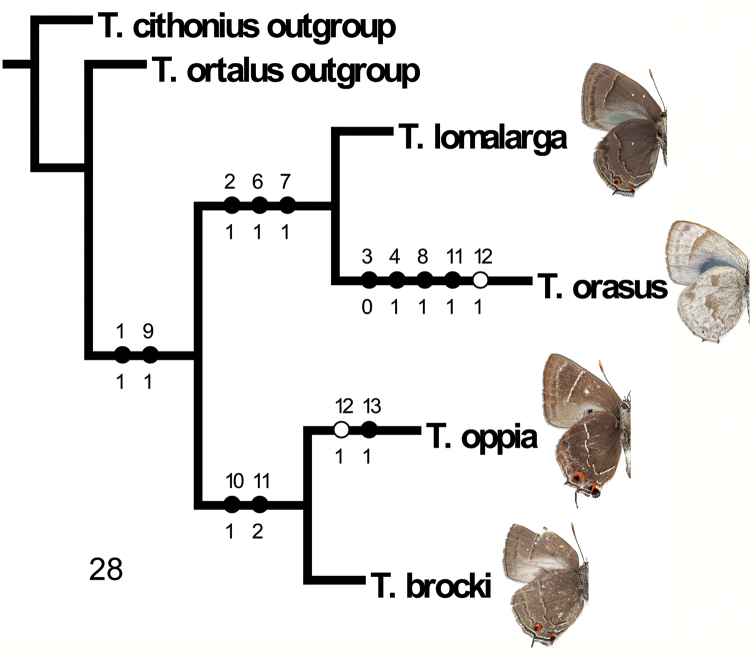
Most parsimonious cladogram for of the *Thereus oppia* species group with unambiguous character state changes (22 steps, CI = 81, RI = 66). Hollow circles are homoplastic changes. Numbers to right of nodes in brackets are bootstrap values. The dorsal forewing scent pad (Character 12) was unambiguously lost twice. See text for further explanation.

(Fig. 26). Costa Rica to the western slope of the Andes in Ecuador. It is allopatric with *Thereus orasus*, its hypothesized phylogenetic sister (Fig. 28). Their ranges overlap in Costa Rica and Panama, but in these countries, *Thereus lomalarga* is recorded below 1100 m and *Thereus orasus* at 1800 m.

#### Habitat.

*Thereus lomalarga* occurs in the great variety of forested habitats. In Central America, it occurs from sea level to lower montane humid forest at 1100 m elevation. In South America, it also occurs at elevations up to 1600 m. Although two females have labels with the elevation range 1375–1700 m, we have since learned from the collectors that they were found in the lower half of this range.

#### Phenology.

Adults in Panama were collected during the dry season without exception, suggesting adult seasonality. However, caterpillars in Colombia were found throughout the year and reared to the adult stage. Perhaps adults of this species are more apparent to collectors during the dry season.

#### Male behavior.

Six males displayed territorial hilltopping behavior from 09:00–09:30 hours during the dry season (February, March 1979) at the top of a small tree on the southwest edge of the summit of Cerro Luisa, Paraíso (9°02'N, 79°37'W), Canal Area, Panama (vouchers in USNM). The longitude on one specimen is incorrectly labeled 79°38'W.

#### Caterpillar food plant.

*Oryctanthus alveolatus* (H.B.K.) Kuijt (Loranthaceae) growing on *Miconia minutiflora* (Bonpl.) DC. Details of the life history will be published elsewhere (Heredia and Robbins in prep.).

#### Remarks.

*Thereus lomalarga* is a peculiar butterfly in that adult females are far more frequently encountered—at least by butterfly collectors—than are adult males. For example, all collected adults from Costa Rica, Colombia, and Ecuador are females. Among collected adults in Panama, females have been found from sea level in the Canal Area to Cerro Campana (at about 850 m along the trail to the summit, Panama Province) to Potrerillos at 1,100 m (Chiriquí Province). In contrast, adult males have been collected only at the top of one small tree on Cerro Luisa in the Canal Area in the dry season in 1979. All other males, including the holotype, were reared from caterpillars. Among 44 reared individuals at the type locality, 27 are males, so the sex ratio among immatures is not biased towards females.

No museum specimens other than those in the type series have been seen by the authors. However, females of *Thereus lomalarga* are “non-descript small gray hairstreaks”, and other specimens may be found in museum collections.

### Thereus oppia

Taxon classificationAnimaliaLepidopteraLycaenidae

(Godman & Salvin, 1887)

Figs 3 , 7 , 11 , 14 , 17 , 21

#### Diagnosis.

The male of *Thereus oppia* is distinguished from *Thereus lomalarga* and *Thereus brocki* by lacking a scent pad on the dorsal forewing (Fig. 3) and from *Thereus orasus* by having a darker gray/brown ventral ground color (Fig. 3). Both sexes differ from the other members of the species complex by lacking dark scaling along the basal edge of the postmedian line on the ventral wings (Fig. 3). *Thereus oppia* has small teeth on the ventral tip of the penis (Fig. 17), in contrast to *Thereus brocki* and *Thereus orasus*.

#### Nomenclature.

We examined a syntype of this species in the BMNH.

#### Distribution and habitat.

*Thereus oppia* occurs from Mexico to Costa Rica at a variety of elevations. Most localities where it occurs appear to be deciduous dry forest. It is allopatric with its sister species, *Thereus brocki*.

#### Male behavior.

Territorial male behavior was observed at Ciudad Valles, SLP, Mexico in the early afternoon (vouchers in RCB), in contrast to the early morning territorial behavior of *Thereus lomalarga*.

#### Adult flower feeding.

Adults of *Thereus oppia* were found nectaring on *Cordia* (Boraginaceae) flowers at two localities in Veracruz, Mexico (vouchers in RCB).

#### Caterpillar food plant.

From Janzen and Hallwachs (2015), a pupa was found 30 April 1993 on *Struthanthus orbicularis* (Kunth) Blume (Loranthaceae) at Sendero Carobonal, Santa Rosa, Area de Conservación Guanacaste, Guanacaste, Costa Rica, latitude 10.77594, longitude -85.65799. An adult male (voucher 93-SRNP-30, deposited USNM) emerged 16 May 1993. As an associated comment on the web site, “red-brown pupa with white markings laterally so that it looks just like a bird turd, sitting on the top of a mistletoe leaf in middle of large plant (this species is a sprawler, vine/shrub); host tree was leafless.” The leafless host tree is the reason that we consider the mistletoe plant on which the pupa was found to be the caterpillar food plant.

### Thereus brocki

Taxon classificationAnimaliaLepidopteraLycaenidae

Robbins, Heredia & Busby sp. n.

http://zoobank.org/458AA4B0-A519-40D3-90BA-9FC7F286DBCE

Figs 4 , 8 , 12 , 18 , 22 , 24

#### Type material.

**Holotype**: ♂ (Fig. 4). [printed and handwritten on white paper] 28 June 1980/25 km. n. e. of Puyo,/Prov. Pastaza, Ecuador/leg. Jim P. Brock [printed on green paper] GENITALIA No./2013: 56♂/R. K. ROBBINS [printed white barcode label] USNM ENT 00181942 [printed label on red paper] Holotype/*Thereus brocki*/Robbins, Busby, & Heredia. Deposited USNM.

#### Paratype

(1♀). **Ecuador**. 1♀ (Fig. 4). Napo, 14 km Tena-Puyo Road, Apuya, 01°06.7'S,77°46.9'W, 600 m, 10 Sep 2010, (RCB).

#### Other specimens

(1♂,2♀). **Colombia**. 1♀. Vaupés, Mitú, 28 Jun 1972 (USNM). **Peru**. 1♂ San Martin, Juanjuí, 7°11'S,77°44'W, 300–400 m, Nov 2011 (MUSM, examined from an image). 1♀. Huánuco, Tingo María, 800 m, 24 Jun 1982 (USNM).

#### Etymology.

This species is named for James Brock of Tucson, Arizona. He collected the holotype and has made numerous contributions to the knowledge and enjoyment of butterflies. The name is a masculine noun in the genitive case.

#### Type locality.

The type locality has been a well-known collecting site for 40 years at about 975 m elevation (noted in Brown 1979 with coordinates 01°20'S,77°55'W, but incorrectly placed in Napo Province). The entrance to this locality is approximately 25 km (measured by a car odometer) from Puyo on the western side of the Puyo-Tena Rd. New metal road markers have been erected which place the entrance between km 26 and km 27. The trees in this location have been selectively logged for decades, and we do not believe much forest remains.

#### Diagnosis and description.

*Thereus brocki* belongs to *Thereus* because it possesses the synapomorphies of the genus (Figs 18, 24). It belongs to the *Thereus oppia* species group (Figs 4, 12). It shares a dorsal forewing scent pad with *Thereus lomalarga* (Figs 6, 8), but differs in having a gray-brown scent patch on the dorsal hindwing without iridescence and in possessing erect piliform setae on the inner margin of the ventral forewing (Fig. 4). The male lacks evident teeth on the dorsal tip of the penis (Fig. 18), which distinguishes it from *Thereus lomalarga* and *Thereus oppia*. The female of *Thereus brocki* is very similar to that of *Thereus lomalarga*, but differs in having more orange-red scales on the ventral hindwing at the anal lobe (Fig. 4). The wing pattern, androconia, and genitalia are illustrated (Figs 4, 8, 12, 18, 22, 24). Forewing size of the holotype male is 0.9 cm and of two females is 1.1 cm and 1.2 cm.

#### Female.

The female paratype of *Thereus brocki* is associated with the male by the shape similarity of the ventral hindwing postmedian line and by their capture approximately 30 km apart. Although the females of *Thereus brocki* and *Thereus lomalarga* illustrated in Figs 2 & 4 would seem to be distinguishable phenotypes, wing pattern variation in the extensive type series of the latter encompasses both phenotypes. For this reason, we restricted the paratype series. We unsuccessfully tried to extract DNA sequences from *Thereus brocki* to confirm the identification of the females.

#### Sexual dimorphism.

Forewing discal cell length in the male of *Thereus brocki* (Fig. 12) is shorter than in the female.

#### Distribution

(Fig. 26). Eastern Colombia to eastern Peru. It is allopatric with its phylogenetic sister, *Thereus oppia*.

#### Habitat.

*Thereus brocki* has been recorded only from wet forest up to about 1,000 m elevation.

#### Remarks.

The holotype and the Peruvian male are the only males in collections, so far as we are aware. The type locality has been a “famous” collecting locality for decades, as noted, so it is somewhat unexpected that the holotype remains the only known Ecuadorian male. It would appear that adult males of *Thereus brocki*, like those of *Thereus lomalarga*, are rarely encountered by insect collectors. Although we have an image of the Peruvian male (discovered late in the publication process), we have not had an opportunity to examine it. It is identified as *Thereus brocki* because it has the shortened forewing discal cell of the holotype and the same male wing secondary sexual traits, except that the erect piliform setae are not visible in the image. For this reason, we exclude it from the type series.

### Nomenclatural checklist – *Thereus oppia* species group

***Thereus orasus*** (Godman & Salvin, 1887) (*Thecla*)

type locality: Guatemala

= *echinita* (Schaus, 1902) (*Thecla*)

type locality: Mexico (VER)

***Thereus lomalarga*** Robbins, Heredia & Busby, sp. n.

type locality: Colombia

***Thereus oppia*** (Godman & Salvin, 1887) (*Thecla*)

type locality: Mexico (VER)

***Thereus brocki*** Robbins, Heredia & Busby, sp. n.

type locality: Ecuador

### Phylogenetic analyses

Based on morphological characters (Table 1) coded in a matrix (Table 2), there was one most parsimonious 22-step equal weight tree (CI = 81 and RI = 66). Furthermore, each implied weight most parsimonious tree with different values for the parameter *K* resulted in one tree, also with the same topology as the most parsimonious equal weight tree. When the data were analyzed with the scent pad omitted (Character 12), the tree topology was the same. In accord with Quental’s (2008) results, unambiguous optimization suggests that the scent pad was lost twice and not regained (Character 12, Fig. 28).

## Discussion

**Systematics.** The genus *Thereus* has been described six times, probably because the genus contains species that possess a variety of androconial organs, wing patterns, wing shapes, and wing sizes. However, *Thereus* is clearly characterized by synapomorphies of the male genitalia, female genitalia, and antennae, as illustrated in this paper. Further, a third of the species have been reared, and with the exception of an undescribed, morphologically aberrant species, all use Loranthaceae as a caterpillar food plant. A genus that is morphologically and biologically homogenous, such as *Thereus*, conveys information, which makes it a useful and predictive classification. For example, it allows us to hypothesize that all *Thereus* species that have not yet been reared, including *Thereus brocki*, eat Loranthaceae.

The wing patterns of the newly described *Thereus lomalarga* and *Thereus brocki* are very similar (Figs 2, 4), for which reason these species might well be thought of as likely sister species. However, the phylogenetic analysis is consistent with the hypothesis that the wing pattern similarities are symplesiomorphies. The opposite situation occurs with the wing patterns of *Thereus orasus* and *Thereus oppia* (Figs 1, 3). Historically, these species were not considered to be close relatives (i.e., Draudt 1919–1920), presumably because their wing patterns are quite different (Figs 1, 3). The phylogenetic analysis makes clear that the wing pattern of *Thereus orasus* is divergent within the *Thereus oppia* species group because of evolutionary wing pattern changes in the ancestor of *Thereus orasus*, not because of a lack of relationship.

**Biology.** Males of the newly described *Thereus lomalarga* and *Thereus brocki* are exceedingly rare, at least in museum collections. For example, adult males of *Thereus lomalarga* have been collected at only one site, despite more than a century of butterfly collecting in Costa Rica, Panama, western Colombia, and western Ecuador. Alternately, immatures of three of the four species in the *Thereus oppia* group are associated with Loranthaceae. Searching for caterpillars on Loranthaceae may be a more efficient way to find males (and females) of these and related species. More generally, it may be the best way to document and study the biology of *Thereus* species.

Erect piliform setae on the ventral forewing that attach to the forewing inner margin (Fig. 14) evolved in the ancestor of *Thereus oppia* and *Thereus brocki* (Fig. 28, Character 10). They occur in no other Eumaeini and are presumably a newly evolved male secondary sexual organ. This evolutionary gain neither increased nor decreased diversification. These piliform setae are superficially similar to those that occur almost universally in the related tribe Deudorigini (Eliot 1973). The evident difference is that the erect setae in *Thereus* are associated with a scent patch on the ventral forewing while those in the Deudorigini are not. It remains an open question whether the genetic pathway by which these erect setae develop is the same in *Thereus* and the Deudorigini.

Quental (2008) postulated that the eumaeine scent pad has been lost evolutionarily many times without being regained, following Dollo’s law. In the *Thereus oppia* species group, two species possess a scent pad and two lack it, and it is reasonable to ask whether the scent pad could have been re-gained evolutionarily in this group. Indeed, cladograms such as (*Thereus oppia* + (*Thereus orasus* + (*Thereus lomalarga* + *Thereus brocki*))) or (*Thereus orasus* + (*Thereus oppia* + (*Thereus lomalarga* + *Thereus brocki*))) would be consistent with the evolutionary loss and re-gain of the scent pad. However, according to the phylogenetic results in this paper, in which the scent pad character was omitted, the scent pad was unambiguously lost twice evolutionarily and not regained. In each case, the species that lost the scent pad is allopatric with its sister species. This result is consistent with previous findings (Quental’s 2008, Robbins et al. 2012) and more generally, with Wiens’ (2001) observations on the evolution of male secondary sexual traits in animals.

## Supplementary Material

XML Treatment for Thereus

XML Treatment for Thereus orasus

XML Treatment for Thereus lomalarga

XML Treatment for Thereus oppia

XML Treatment for Thereus brocki
